# Feasibility and Safety on Left Bundle Branch Area Pacing With Standard Stylet‐Driven Lead: ACHIEVE‐SYNC Multicenter Prospective Observational Cohort Study

**DOI:** 10.1002/joa3.70345

**Published:** 2026-05-02

**Authors:** Ga‐In Yu, So‐Ryoung Lee, Tae‐Hoon Kim, Min Soo Cho, Daehoon Kim, Hee Tae Yu, Jung‐Myung Lee, Jae‐Sun Uhm, Eue‐Keun Choi, Jun Kim, Boyoung Joung, Hui‐Nam Pak, Moon‐Hyoung Lee

**Affiliations:** ^1^ Division of Cardiology, Gyeongsang National University Changwon Hospital Gyeongsang National University College of Medicine Changwon Republic of Korea; ^2^ Division of Cardiology, Seoul National University Hospital Seoul National University College of Medicine Seoul Republic of Korea; ^3^ Division of Cardiology, Severance Hospital Yonsei University College of Medicine Seoul Republic of Korea; ^4^ Division of Cardiology, Asan Medical Center Ulsan University College of Medicine Seoul Republic of Korea; ^5^ Division of Cardiology, Sahmyook Medical Center, Seoul Hospital Sahmyook University College of Medicine Seoul Republic of Korea

**Keywords:** conduction system pacing, left bundle branch area pacing, stylet‐driven pacing lead

## Abstract

**Background:**

Right ventricular pacing increases the risk of dyssynchrony, which raises the need for more physiological pacing strategies. Left bundle branch area pacing (LBBAP) has emerged, with most early studies utilizing lumen‐less pacing leads. The feasibility and safety of LBBAP using conventional stylet‐driven pacing lead (SDL) have been reported. We present prospective multicenter data, particularly in Asian clinical settings.

**Methods:**

The ACHIEVE‐SYNC pilot study was a multicenter prospective observational cohort study conducted across several tertiary hospitals in South Korea. Patients with standard indications for pacemaker implantation underwent LBBAP using a 5.6Fr SDL with an extendable screw. Procedural success rate and LBBAP‐related complications were evaluated. Pacing parameters, electrocardiographic features, and echocardiographic outcomes were assessed up to 12 months after implantation.

**Results:**

LBBAP using SDL was successful in 100 of 101 (99.0%) patients. LBBAP lead‐related adverse event occurred in 1 case (0.99%), which was lead dislodgement. The median pacing threshold at 12‐month follow‐up was 0.8 [0.7–1.0] V at 0.5 ms. At 12‐month follow‐up, the mean QRS duration changed from 119.2 ± 28.6 ms to 131.2 ± 23.8 ms. The mean left ventricular ejection fraction was 61.3% ± 8.5% before the procedure and 61.3% ± 7.5% at 12‐month follow‐up.

**Conclusions:**

In this prospective multicenter registry, LBBAP using SDL demonstrated a near‐complete procedural success rate and a high incidence of confirmed Left bundle branch capture. Lead performance remained stable during long‐term follow‐up, and adverse events were rare, supporting the safety and technical feasibility of this approach in routine clinical practice.

## Graphical abstract

In the prospective multicenter study, left bundle branch area pacing using stylet‐driven pacing leads demonstrated a high procedural success rate and showed stable lead performance during long‐term follow‐up.

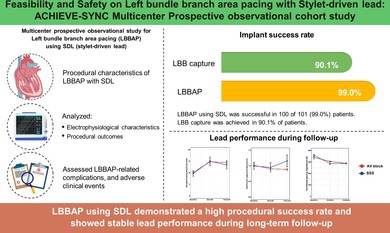

## 1 Introduction

Conventional right ventricular pacing (RVP) in patients with bradyarrhythmia leads to cardiac dyssynchrony and increases the risk of poor outcomes such as pacemaker‐induced cardiomyopathy and heart failure (HF) [1, 2], which has led to the development of conduction system pacing (CSP) and has become the basis for His bundle pacing (HBP) [3]. However, although HBP is an ideal strategy for physiological pacing, it has the limitations of being difficult to perform because the His bundle is a small area requiring high pacing output in long‐term follow‐up [4]. In the midst of this, left bundle branch area pacing (LBBAP) as a physiological CSP modality has been proposed [5].

Compared to HBP, LBBAP allows operators to adapt to the procedure faster and shows a stable lead pacing threshold over the long term [6, 7]. The positive results have been proven through major large‐scale studies [8, 9]. Most early experiences of LBBAP have been achieved using a lumen‐less pacing lead (LLL) [5, 10]. However, the safety and efficiency of LBBAP with conventional stylet‐driven lead (SDL), which is used in traditional RVP, has also been demonstrated in previous studies [11, 12]. LBBAP using SDLs is noninferior to that using LLLs in terms of implantation success, and there were no differences in procedural and electrophysiological characteristics [13]. Being able to perform LBBAP using existing conventional SDL has clear benefits, including accessibility to the procedure.

Considering the smaller cardiac anatomy and thinner septum of Asian patients [14], the feasibility of deep septal access using the larger‐diameter SDL compared to LLL warrants dedicated investigation. While major studies have established the role of LBBAP using LLL in Asian populations, evidence regarding SDL has also begun to emerge with recent observational reports [15, 16]. However, prospective multicenter data specifically focusing on the feasibility and safety of the SDL approach remain limited. Given the potential anatomical considerations in Asian patients, providing robust clinical evidence through a structured prospective registry is essential to validate the broader applicability and long‐term stability of LBBAP using SDL. Therefore, this study aimed to investigate the procedural and long‐term clinical outcomes of SDL‐mediated LBBAP in a multicenter Asian cohort.

## 2 Methods

### 2.1 Study Population

The ACHIEVE‐SYNC study (FeAsibility and Safety of left bundle branCH pacIng with standard stylEt‐driVEn lead to minimize pacing‐induced ventricular dysSYNChrony and heart failure progression) was a prospective, observational, multicenter registry conducted across four tertiary centers in South Korea. Between 2021 and 2022, a total of 101 patients who underwent LBBAP using SDL were enrolled. These patients were selected from among those with standard indications for permanent pacemaker implantation. Inclusion criteria were as follows: (a) an anticipated ventricular pacing burden ≥ 40%, or (b) a baseline wide QRS duration, defined as > 130 ms in patients with left bundle branch block (LBBB), or > 150 ms in those without LBBB. Exclusion criteria included: (1) those younger than 18 years of age; (2) those with indications eligible for implantable cardioverter‐defibrillator or cardiac resynchronization therapy; (3) those who have valvular heart disease, including a past history of undergoing prosthetic valve surgery of the tricuspid valve; and (4) those who have a history of myocardial infarction involving the ventricular septum.

The study protocol was approved by the Institutional Review Boards of all participating centers, including the coordinating center at Yonsei University Health System (4–2021‐1040). The study was conducted in accordance with the principles of the Declaration of Helsinki.

### 2.2 Definition of LBBAP and LBBP

To confirm successful LBBAP, 12‐lead surface ECG and intracardiac electrograms were continuously monitored with an electrophysiology recording system during the procedure. LBBAP was defined as pacing within the left bundle branch area, encompassing both left bundle branch pacing (LBBP) and left ventricular septal pacing (LVSP). While advancing the SDL into the target area, if the LBBB pattern gradually diminished and the right bundle branch block (RBBB) pattern (Qr, qR, or rSR) in V1 was seen during unipolar pacing, it was considered a success for LBBAP regardless of the confirmation of LBB capture. Accordingly, LBBAP includes LVSP, as well as nonselective LBBP and selective LBBP. LBBP was defined as successful LBB capture, based on established criteria from prior studies and guidelines [17, 18].

The definitions and criteria applied in this study were based on the available evidence at the time of study design and enrollment (2021) and broadly align with the principles later formalized in recent consensus statements, including the EHRA clinical consensus statement. Left bundle branch (LBB) capture was considered successful when one or more of the following findings were identified during unipolar pacing, in addition to a RBBB pattern in lead V1: (1) QRS transition from nonselective LBB capture to left ventricular septal capture with an abrupt increase in V6 R‐wave peak time (V6 RWPT) of > 10–15 ms during threshold testing; (2) QRS transition from nonselective to selective LBB capture near the capture threshold, with an abrupt increase in V1 RWPT of > 10 ms; (3) A short and constant stimulus‐to‐left ventricular activation time (stim‐LVAT), with the shortest stim‐LVAT < 75 ms in non‐LBBB and < 85 ms in LBBB; (4) Change in QRS morphology from nonselective LBB capture to either left ventricular septal capture or selective LBB capture during programmed stimulation via the pacing lead.

In cases where LBB capture was not confirmed, but the pacing lead was observed to traverse the right ventricular septum and capture the left ventricular septal myocardium, the pacing was defined as LVSP. Such cases were also classified under LBBAP, reflecting the inclusive procedural definition used in this study [19]. In this study, a pacing capture threshold of ≤ 2.0 V at 0.4–0.5 ms was required to define successful LBBAP or LVSP, consistent with the EHRA clinical consensus statement and established procedural standards. Examples of intracardiac electrograms obtained during the LBBAP procedure are presented in Figure S1.

### 2.3 LBBAP Lead Implantation Using SDL

LBBAP was performed using a 5.6‐Fr SDL with an extendable screw (Solia S60, Biotronik SE & Co., Berlin, Germany). The lead was prepared in the manner previously described in the report [20]. To sum up, the screw was extended by turning the outer pin clockwise 10–12 times, followed by an additional five times of clockwise turns of the outer pin using the stylet guide tool delivered with the lead to avoid partial unwinding of the screw. The SDL is delivered through a pre‐shaped sheath (Selectra 3D, Biotronik, SE & Co KG, Berlin, Germany) because it properly targets the pacing site and maintains sufficient backup during lead implantation.

Methods for finding the optimal spot for attempting the procedure were using fluoroscopy to predict the location of the LBB area based on the His area or dividing the right ventricle (RV) into nine segments and attempting the procedure at the most likely location [21, 22]. In the former method, after the His area was confirmed using a His catheter during the electrophysiology study, LBBAP was attempted by targeting a position 1–2 cm from that position in the direction of the right ventricular apex in the right anterior oblique (RAO) view on fluoroscopy (Figure S2A) [21, 23]. In the latter method, in the RAO view on fluoroscopy, the RV was divided into nine segments, and the procedure was performed targeting the high‐ and mid‐septal areas (Figure S2B) [24, 25].

To maintain lead tension, the pacing lead was screwed to the interventricular septum from the right side to the left side while ensuring the stylet was inserted fully. Subsequently, the pacing lead was advanced by fast rotation 5–10 times to overcome the septal resistance and keep the stylet in the pacing lead until the final position is reached [20, 26]. A sudden decrease of lead impedance, amplitude of the sensed R‐wave, and/or loss of capture indicated that the helix of the lead had entered the chamber of the left ventricle (LV), e.g., LV perforation. If this occurred, the pacing lead should be rotated back and relocated.

### 2.4 Follow‐Up and Study Outcome

Data on patients were collected prior to pacemaker implantation, including clinical information, baseline demographic characteristics, medication history, 12‐lead ECG and 24‐h Holter ECG data, treadmill test data, and transthoracic echocardiography (TTE) findings. Analyses of 12‐lead ECG, TTE, and lead parameters recorded on the device were performed at 3, 6, and 12 months after LBBAP implantation using SDL. Immediately after lead implantation, both unipolar and bipolar values were measured for lead parameters, and only bipolar data were checked during follow‐up. Lead parameters included the amplitude of the sensed R wave, ventricular capture threshold, and ventricular pacing impedance.

Clinical events and LBBAP lead‐related events were evaluated at each patient visit. Clinical events included all‐cause death, hospitalization due to HF, major adverse cardiac events, newly detected atrial fibrillation, and cerebral infarction. LBBAP lead‐related events included death related to LBBAP; permanent loss of LBBAP lead function; need for lead revision, reposition, replacement, or explant; and prolonged hospitalization (48 h) related to the procedure.

### 2.5 Statistical Analysis

Descriptive statistics were used to organize and interpret baseline characteristics and comorbidities of the patients. Categorical variables are reported as frequencies (percentages). Continuous variables are reported as mean ± standard deviation or median (interquartile range). Categorical variables were compared using Fisher's exact test or Pearson's chi‐square test, whereas continuous variables were compared using the Student's *t*‐test and the Wilcoxon rank‐sum test.

All tests were two‐tailed, with a *p*‐value < 0.05 being considered significant. The statistical analyses were performed using R programming version 4.0.3 (The R Foundation for Statistical Computing, Vienna, Austria).

## 3 Results

### 3.1 Baseline Characteristics

A total of 101 patients who underwent LBBAP (median age 74.0 [64.0–80.0] years, 41.6% female) were included in this study. The total success rate of lead implantation in LBBAP was 99.0% (100/101) for all patients. LBBP (successful LBB capture confirmed) and LVSP among all successful LBBAPs were 91.0% (91/100) and 9.0% (9/100), respectively. Pacing indication was sick sinus syndrome (SSS) in 13.9% (14 of 101) and atrioventricular block (AVB) in 86.1% (87 of 101). 10.2% of all patients (9 of 101) had a history of HF with left ventricular ejection fraction less than 50%. Baseline characteristics were summarized in Table 1.

**TABLE 1:** Baseline characteristics.

	All (*N* = 101)	LBBAP		LBBAP failure (*n* = 1)	*P*‐value
		LBBP^a^ (*n* = 91)	LVSP (*n* = 9)		
Demographics					
Age, years	74.0 [64.0–80.0]	75.0 [64.0–80.0]	68.0 [64.0–70.0]	60.0 [60.0–60.0]	0.165
Female sex	42 (41.6%)	40 (44.0%)	2 (22.2%)	0 (0.0%)	0.315
Body mass index, kg/m^2^	24.1 [21.6–26.7]	24.2 [22.0–26.7]	23.4 [20.1–25.6]	22.5 [22.5–22.5]	0.474
Indication for LBBAP					0.287
Sick sinus syndrome	14 (13.9%)	11 (12.1%)	2 (22.2%)	1 (100.0%)	
AV block	87 (86.1%)	80 (87.9%)	7 (77.8%)	0 (0.0%)	
Second‐degree AV block	17 (16.8%)	16 (17.6%)	1 (11.1%)	0 (0.0%)	
High degree AV block	17 (16.8%)	15 (16.5%)	2 (22.2%)	0 (0.0%)	
Complete AV block	53 (52.5%)	49 (53.8%)	4 (44.4%)	0 (0.0%)	
Baseline QRS morphology					0.657
Narrow QRS	45 (45.9%)	43 (48.9%)	1 (11.1%)	1 (100.0%)	
RBBB	26 (26.5%)	22 (25.0%)	4 (44.4%)	0 (0.0%)	
LBBB	14 (14.3%)	12 (13.6%)	2 (22.2%)	0 (0.0%)	
Bifascicular block	6 (6.1%)	5 (5.7%)	1 (11.1%)	0 (0.0%)	
Trifascicular block	0 (0.0%)	0 (0.0%)	0 (0.0%)	0 (0.0%)	
IVCD	7 (7.1%)	6 (6.8%)	1 (11.1%)	0 (0.0%)	
Paced rhythm	0 (0.0%)	0 (0.0%)	0 (0.0%)	0 (0.0%)	
Comorbidities					
Hypertension	58 (58.0%)	51 (56.7%)	7 (77.8%)	0 (0.0%)	0.236
Diabetes mellitus	35 (34.7%)	32 (35.2%)	3 (33.3%)	0 (0.0%)	0.760
Congestive heart failure	7 (7.0%)	4 (4.4%)	3 (33.3%)	0 (0.0%)	0.005
Atrial fibrillation	17 (17.5%)	12 (13.8%)	4 (44.4%)	1 (100.0%)	0.007
Ischemic stroke or TIA	8 (7.9%)	8 (8.8%)	0 (0.0%)	0 (0.0%)	0.620
Medication history					
Oral anticoagulant	19 (18.8%)	13 (14.3%)	6 (66.7%)	0 (0.0%)	< 0.001
Aspirin or P2Y12 inhibitor	25 (24.8%)	24 (26.4%)	1 (11.1%)	0 (0.0%)	0.507
ACE inhibitors or ARB	12 (11.9%)	10 (11.0%)	2 (22.2%)	0 (0.0%)	0.570
Beta‐blockers	7 (6.9%)	6 (6.6%)	1 (11.1%)	0 (0.0%)	0.846
Calcium channel blockers	38 (37.6%)	35 (38.5%)	3 (33.3%)	0 (0.0%)	0.704
Statin	40 (39.6%)	35 (38.5%)	5 (55.6%)	0 (0.0%)	0.435
Echocardiographic parameters					
LVEF, %	61.3 ± 8.5	62.3 ± 7.6	53.2 ± 11.6	NA^b^	0.002
E/E'	12.5 [10.0–19.5]	12.0 [10.0–20.0]	13.8 [11.5–15.7]	NA^b^	0.554

*Note:* Values are presented as mean ± standard deviation, median [Q1–Q3], or *n* (%).

Abbreviations: ACE, angiotensin‐converting enzyme; ARB, angiotensin II receptor blocker; AV block, atrioventricular block; IVCD, intraventricular conduction delay; LBBAP, left bundle branch area pacing; LBBB, left bundle branch block; LBBP, left bundle branch pacing; LVEF, left ventricular ejection fraction; LVSP, left ventricular septal pacing; RBBB, right bundle branch block; SSS, sick sinus syndrome; TIA, transient ischemic attack.

^a^ When LBB capture was observed, it was confirmed to be LBBP.

^b^ NA: Not applicable.

### 3.2 Procedural Outcomes and LBBAP Characteristics

During the LBBAP procedure using SDL, LBB potentials were observed in 18.8% (19/101) of patients, and the median Stim‐LVAT was 75.4 ± 11.8 ms. Immediately after the lead implantation, lead parameters were evaluated in both bipolar and unipolar mode. Concretely, the median bipolar and unipolar ventricular capture threshold was 1.0 [0.7–1.3] volt (V) at 0.4 ms and 1.1 [0.7–1.5] V at 0.4 ms, respectively. The mean baseline QRS duration was 119.2 ± 28.6 ms, and the mean paced QRS duration immediately after the lead implantation was 126.9 ± 21.2 ms, without a significant difference between the SSS and AVB groups. The electrophysiological characteristics of LBBAP are summarized in Table 2.

**TABLE 2:** Electrophysiological characteristics of LBBAP using SDL.

	All (*N* = 101)	SSS (*N* = 14)	AV block (*N* = 87)	*P*‐value
Electrophysiological characteristics				
Observed LBB potential	19 (18.8%)	3 (21.4%)	16 (18.4%)	1.000
Stim‐LVAT, ms	75.4 ± 11.8	78.9 ± 12.6	74.9 ± 11.7	0.270
Pacing parameters, bipolar				
Sensed R wave amplitude, mV	10.1 ± 3.5	9.7 ± 4.4	10.2 ± 3.3	0.704
Ventricular capture threshold, V	1.0 [0.7–1.3]	0.9 [0.7–1.2]	1.0 [0.7–1.3]	0.842
Ventricular pacing impedance, Ω	716.1 ± 112.3	683.7 ± 114.3	720.9 ± 111.9	0.267
Pacing parameters, unipolar				
Sensed R wave amplitude, mV	10.4 ± 3.6	9.7 ± 2.3	10.5 ± 3.8	0.481
Ventricular capture threshold, V	1.1 [0.7–1.5]	1.0 [0.8–1.2]	1.1 [0.7–1.4]	0.578
Ventricular pacing impedance, Ω	643.0 [483.5–795.0]	668.0 [550.0–750.0]	636.0 [470.0–800.0]	0.816
QRS duration				
Baseline QRS duration, ms	114.0 [96.0–140.0]	126.0 [96.0–154.0]	112.0 [95.0–139.0]	0.608
Post LBBAP QRS duration, ms	126.9 ± 21.2	127.3 ± 29.4	126.8 ± 20.4	0.953

*Note:* Values are presented as mean ± standard deviation, median [Q1–Q3], or *n* (%).

Abbreviations: AV, atrioventricular; ms, millisecond; mV, millivolt; LBB, left bundle branch; LBBAP, left bundle branch area pacing; SDL, stylet‐driven pacing lead; SSS, sick sinus syndrome; Stim‐LVAT, stimulus to left ventricular activation time; V, volt; Ω, ohm.

The final locations of LBBAP were left bundle branch or left septal fascicle (77.5%), left posterior fascicle (14.6%), and left anterior fascicle (7.9%). In all patients, fluoroscopy time was 10.6 [7.7–16.0] min, and procedure time was 60.0 [44.0–70.0] min. The procedural outcomes in the participants are summarized in Table 3.

**TABLE 3:** Outcomes of LBBAP using SDL.

	All (*N* = 101)	SSS (*N* = 14)	AV block (*N* = 87)	*P*‐value
Success of LBBAP				0.030
LBBP	91 (90.10%)	11 (78.57%)	80 (91.95%)	
LVSP	9 (8.91%)	2 (14.29%)	7 (8.05%)	
RVP	1 (0.99%)	1 (7.14%)	0 (0.0%)	
Final location of LBBAP pacing lead				0.342
LB trunk or left septal fascicle	69 (77.5%)	10 (90.9%)	59 (75.6%)	
Left anterior fascicle	7 (7.9%)	1 (9.1%)	6 (7.7%)	
Left posterior fascicle	13 (14.6%)	0 (0.0%)	13 (16.7%)	
Procedural characteristics				
Number of attempts, n	2.0 [1.0–2.0]	2.0 [1.0–2.0]	2.0 [1.0–2.0]	1.000
Fluoroscopy time, min	10.6 [7.7–16.0]	13.8 [9.0–22.1]	10.5 [7.6–15.0]	0.088
Procedure time, min	60.0 [44.0–70.0]	66.5 [45.0–75.0]	58.0 [43.5–70.0]	0.578
Adverse event				
LBBAP lead‐related event	1 (1.0%)	0 (0.0%)	1 (1.2%)	1.000
Clinical event	2 (2.0%)	0 (0.0%)	2 (2.3%)	1.000

*Note:* Values are presented as mean ± standard deviation, median [Q1–Q3], or *n* (%).

Abbreviations: AV, atrioventricular; LB, left bundle; LBBAP, left bundle branch area pacing; LBBP, left bundle branch pacing; LVSP, left ventricular septal pacing; RVP, right ventricular pacing; SDL, stylet‐driven pacing lead; SSS, sick sinus syndrome.

### 3.3 Long‐Term Outcomes of LBBAP Using SDL

In the one‐year follow‐up of patients who underwent LBBAP using SDL, LBBAP lead‐related adverse events occurred in 1.0% (1 of 101) of patients. The event involved LBBAP lead dislodgement identified the day after implantation, and lead revision was successfully performed. Throughout the follow‐up period, two patients experienced clinical adverse events: One case of HF hospitalization and one case of cerebral infarction.

**FIGURE 1:**
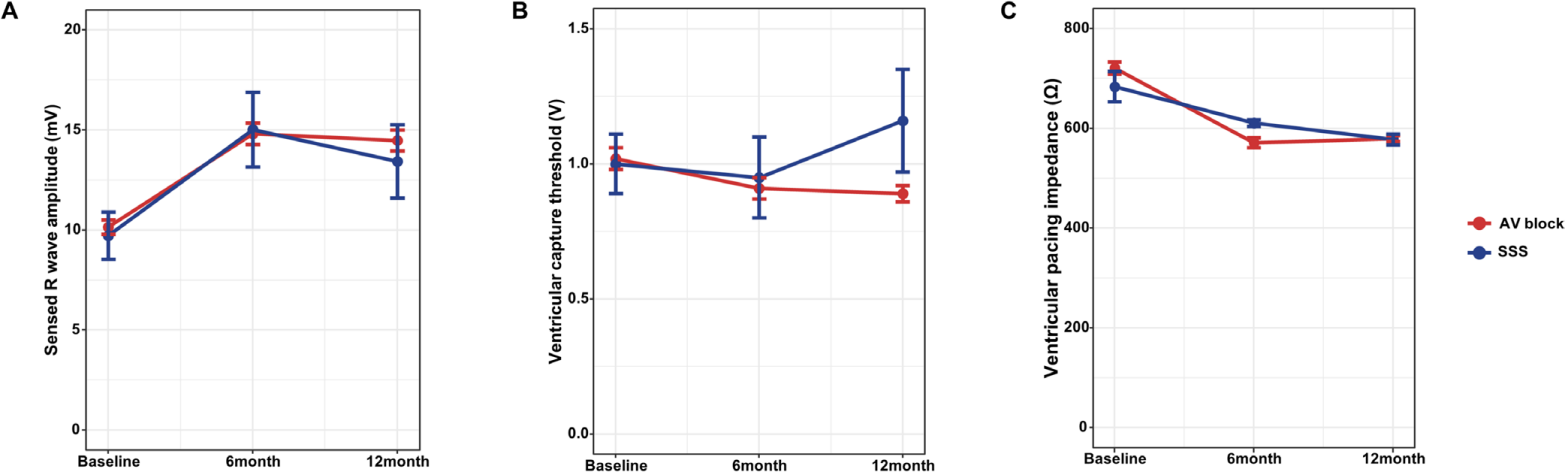
Pacing parameters of LBBAP lead. The amplitude of the sensed R wave (A), ventricular capture threshold (B), and ventricular pacing impedance (C) at baseline, 6 months, and 12 months, respectively. LBBAP, left bundle branch area pacing; AVB, atrioventricular block; SSS, sick sinus syndrome.

Lead parameters of LBBAP using SDL were evaluated in bipolar mode at 3, 6, and 12 months after LBBAP lead implantation (Figure 1). The median amplitude of the sensed R wave was 13.5 [10.0–18.2], 14.8 [10.6–18.7], and 14.0 [10.2–17.9] mV at 3, 6, and 12 months, respectively. The median ventricular capture threshold was 0.8 [0.7–0.9], 0.9 [0.7–1.0], and 0.8 [0.7–1.0] V, respectively, at each visit. The median ventricular pacing impedance was 585.0 [546.0–624.0], 585.0 [546.0–624.0], and 565.0 [546.0–604.0] ohms (Ω), respectively, at each visit.

**FIGURE 2:**
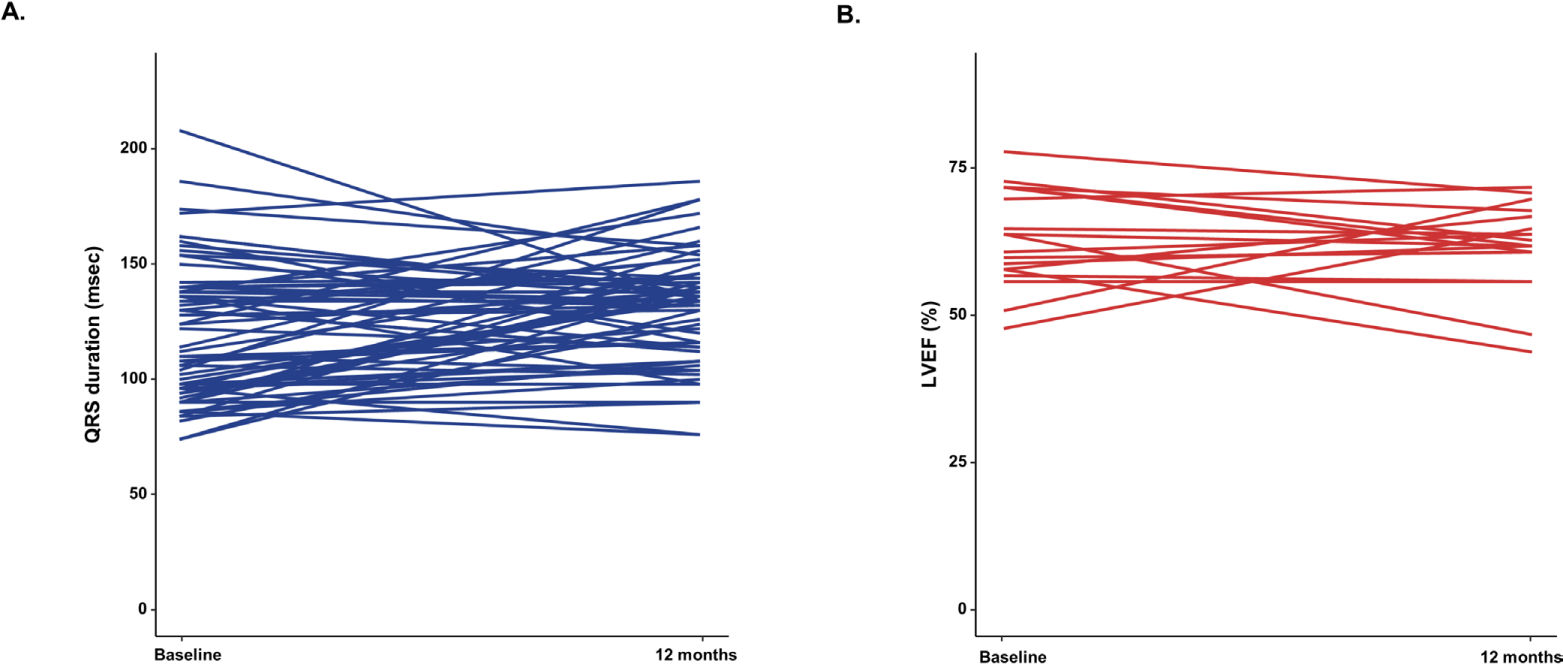
Changes in QRS duration and left ventricular systolic function 12 months after LBBAP. The changes in QRS duration (A) and left ventricular systolic function (B) 12 months after LBBAP. LBBAP, left bundle branch area pacing; LVEF, left ventricular ejection fraction.

At 12‐month follow‐up, the mean QRS duration changed from 119.2 ± 28.6 ms to 131.2 ± 23.8 ms. The mean difference was 10.9 (3.6–18.2) ms, *p*‐value = 0.004. There was no difference in paced QRS duration at 1, 3, 6, and 12 months of serial follow‐up (*p* for interaction = 0.322). Changes in paced QRS duration by baseline QRS morphology during the follow‐up period are presented in Table S1. There was no decline in LV systolic function during the follow‐up period. The mean LVEF before the procedure was 61.3% ± 8.5%, and 61.3% ± 7.5% at 12‐month follow‐up. The mean difference was 1.1 (−3.5–5.6)%, *p*‐value = 0.634. The cardiac synchrony outcome is presented in Figure 2.

## 4 Discussion

Cardiac pacing is an essential therapeutic strategy for bradyarrhythmia. However, traditional RV apical pacing causes electrical and mechanical dyssynchrony, which can lead to poor clinical outcomes such as pacemaker‐induced cardiomyopathy and HF [1, 2]. With this background, physiological CSP has emerged, and HBP has been considered the most ideal physiological pacing. Although HBP is an optimal modality for physiological pacing, the His bundle is an anatomically very narrow area, making the procedure difficult and requiring high pacing output in long‐term follow‐up [4, 27]. In the midst of this, LBBAP, which overcomes the shortcomings of HBP, has been implemented as an alternative strategy for physiological pacing [5, 6]. The long‐term safety of LBBAP has been proven, and the rationale for the procedure has been established [7, 9]. In addition, LBBAP has been proven to be effective and safe in patients with HF and applied to cardiac resynchronization therapy [28, 29, 30].

Most of the initial experiences with LBBAP and related major studies were performed using LLL [5, 10]. However, the LBBAP procedure using SDL with an extendable screw has already been established [10, 11]. LBBAP with conventional SDL used in existing RVP has advantages over LBBAP with LLL, which expands the versatility of LBBAP by providing additional options for procedure tools. First, since SDL is the standard equipment for conventional RVP, it significantly enhances the accessibility of LBBAP for implanters who are already proficient in conventional methods, without requiring them to master an entirely new lead system. Second, SDL provides superior procedural flexibility; in cases where LBBAP cannot be achieved, the same lead can be immediately repurposed for conventional RVP. Third, the use of a stylet provides additional mechanical support and pushability, which can be advantageous for penetrating the interventricular septum in patients with dense septal scarring or increased resistance. Furthermore, it is expected that expanding the use of LBBAP can facilitate the learning curve of physicians [20]. Similarly, many studies on LBBAP using SDL have been published, with safety and benefits being proven, but data in Asian populations remain limited.

In this study, we sought to evaluate the long‐term outcomes of LBBAP using SDL in the Asian population. For this purpose, ECG, TTE, and LBBAP lead parameters were checked at 3, 6, and 12 months after the procedure at the clinic. Appropriate feasibility was observed through the high success rate of the procedure and stable lead parameters immediately after LBBAP using SDL, and long‐term stability was confirmed during the one‐year follow‐up period. Despite a slight, statistically significant increase in the mean QRS duration at 12 months, the achieved QRS duration (131.2 ± 23.8 ms) remained clinically acceptable for patients with standard pacing indications. Although the mean paced QRS duration at 12 months (131.2 ± 23.8 ms) showed a statistical increase compared to baseline, this finding should be interpreted in the context of measurement methodology. Unlike native QRS, paced QRS is measured from the pacing artifact (stimulus), thereby including the latency period between the stimulus and the onset of depolarization. More importantly, our serial analysis revealed that the paced QRS duration remained stable from 1 month to 12 months post‐implantation (p for trend = 0.322). This lack of progressive widening argues against late lead complications, such as micro‐dislodgement or gradual loss of capture, and supports the long‐term stability of the lead‐tissue interface in LBBAP using SDL.

Additionally, all adverse events related and unrelated to LBBAP lead were evaluated. LBBAP lead dislodged in one case, but revision was performed in the same manner without recurrence. There were no significant differences between the SSS and AVB groups in all measured values and clinical events. This study is meaningful in that it confirms the long‐term procedural safety of LBBAP using SDL in the Asian population and provides significant evidence for more in‐depth trials. Although the present prospective multicenter registry was conducted in Asian centers, the fundamental anatomical challenges of conduction system pacing and the mechanical benefits of SDL for LBBAP are universal. Therefore, our findings provide robust, generalized evidence supporting the safety, feasibility, and long‐term reliability of SDL‐based LBBAP, suggesting that this approach can be widely integrated into contemporary clinical practice across diverse global populations.

### 4.1 Study Limitations

This study has some limitations. First, this was an observational, non‐randomized study. However, as a prospective multicenter trial that established a long‐term stable outcome, it lays the foundation for a future randomized study. Second, as a pilot study with a relatively small sample size, the generalizability of the findings is limited. Third, one of the LBB capture criteria used in this study—an absolute V6 RWPT threshold—may lack specificity, as this parameter can occasionally be satisfied even in the absence of true LBB capture. This limitation could partially explain the relatively high LBB capture rate observed in this study. The V6‐V1 interpeak interval, which is highlighted in recent consensus statements as a specific marker for LBB capture, was not included in our analysis because it was not a standard criterion at the time of study design. Our diagnosis relied primarily on QRS transition and stim‐LVAT; therefore, the confirmed LBB capture rate should be interpreted in the context of these evolving diagnostic criteria. Finally, patients with prior septal myocardial infarction, significant valvular disease, or indications for cardiac resynchronization therapy were excluded. This may have favored patients with relatively preserved septal anatomy and may have contributed to selection bias. Consequently, our findings may not be directly generalizable to patients with complex structural heart disease, and future studies involving broader patient populations are warranted.

## 5 Conclusion

In this prospective multicenter pilot study, LBBAP using SDL demonstrated a near‐perfect procedural success rate (99.0%) and a high rate of confirmed LBB capture. Lead performance remained stable over one year of follow‐up, with low complication rates. These findings suggest that LBBAP using SDL is not only feasible but also effective and safe in routine clinical practice. Finally, the LBB capture rate is higher than in previous studies. This is because, according to the design of this study, even if only stim‐LVAT met the criteria, it was considered LBB capture. Furthermore, based on recently established LBBAP criteria, large‐scale, randomized studies are warranted to evaluate the long‐term clinical benefits of this approach and to better define patient populations who may derive the greatest advantage from this pacing modality.

## Funding

This work was supported by Biotronik, FF075. This study was supported by a research grant from Biotronik. The sponsor had no role in the design of the study, data collection, analysis, interpretation, or manuscript writing. The authors affirm that the results and conclusions presented are independent and transparently reported.

## Ethics Statement

The institutional committee (Domain Specific Review Board) has approved this study.

## Consent

Informed consent was taken from patients.

## Conflicts of Interest

The authors declare no conflicts of interest other than the funding provided by Biotronik, which is fully disclosed in the Funding Statement.

## Supporting information


**Figure S1:** Intracardiac electrograms during LBBAP.
**Figure S2:** Methods for finding the optimal site for LBBAP.
**Table S1:** Trends in paced QRS duration according to baseline QRS morphology.

## Data Availability

The data that support the findings of this study are available from the corresponding author upon reasonable request.
