# Supplementary material for: Feasibility and Safety on Left Bundle Branch Area Pacing With Standard Stylet‐Driven Lead: ACHIEVE‐SYNC Multicenter Prospective Observational Cohort Study
Source: J Arrhythm. 2026 May 2;42(3):e70345. doi: 10.1002/joa3.70345 (PMC13135173; doi:10.1002/joa3.70345)
Supplement: Supplementary file 1 — Figure S1: Intracardiac electrograms during LBBAP. Figure S2: Methods for finding the optimal site for LBBAP. Table S1: Trends in paced QRS duration according to baseline QRS morphology. [file JOA3-42-e70345-s001.docx]

**SUPPLEMENTAL MATERIAL**

**Supplementary Figure S1.** Intracardiac electrograms during LBBAP

**
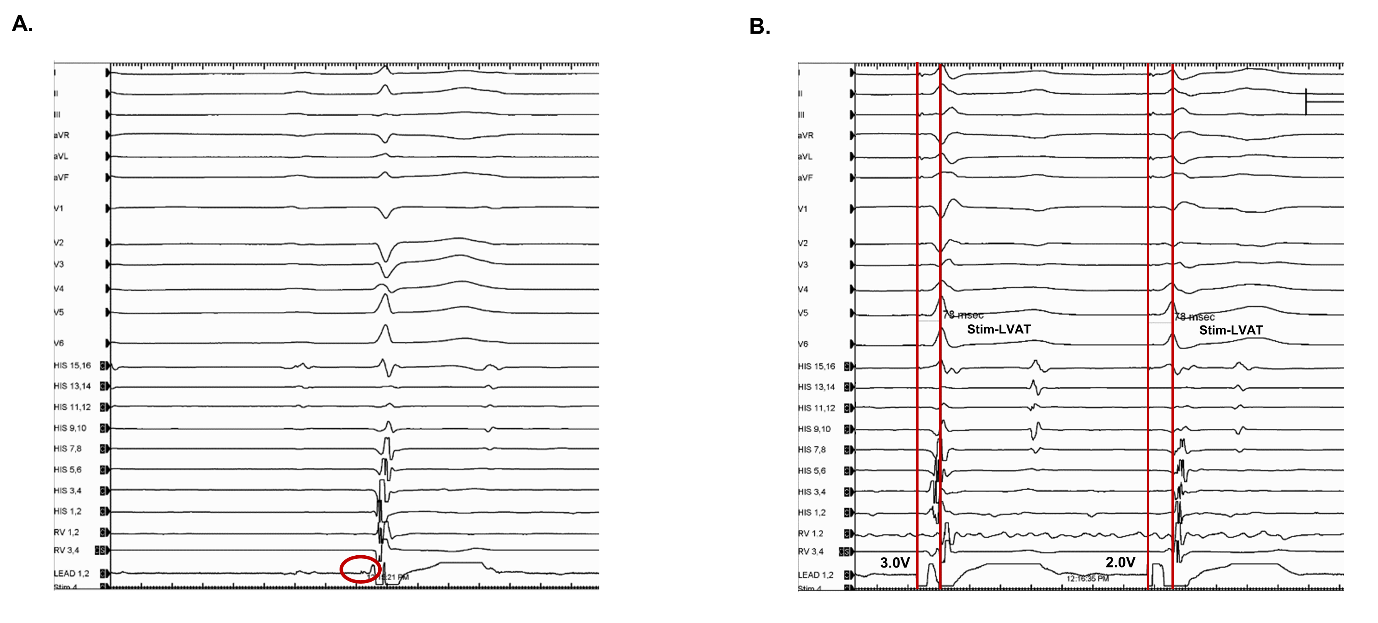
**

LBB potential is shown (A). Stim-LVAT as measured in V5-V6 was <75–85 ms. After further advancement of the lead tip, a transition from nonselective LBB capture (at 3.0 V) to selective LBB capture (at 2.0 V) is observed (B).

LBB, left bundle branch; LBBAP, left bundle branch area pacing; Stim-LVAT, stimulus to left ventricular activation time.

**Supplementary Figure S2.** Methods for finding the optimal site for LBBAP

**
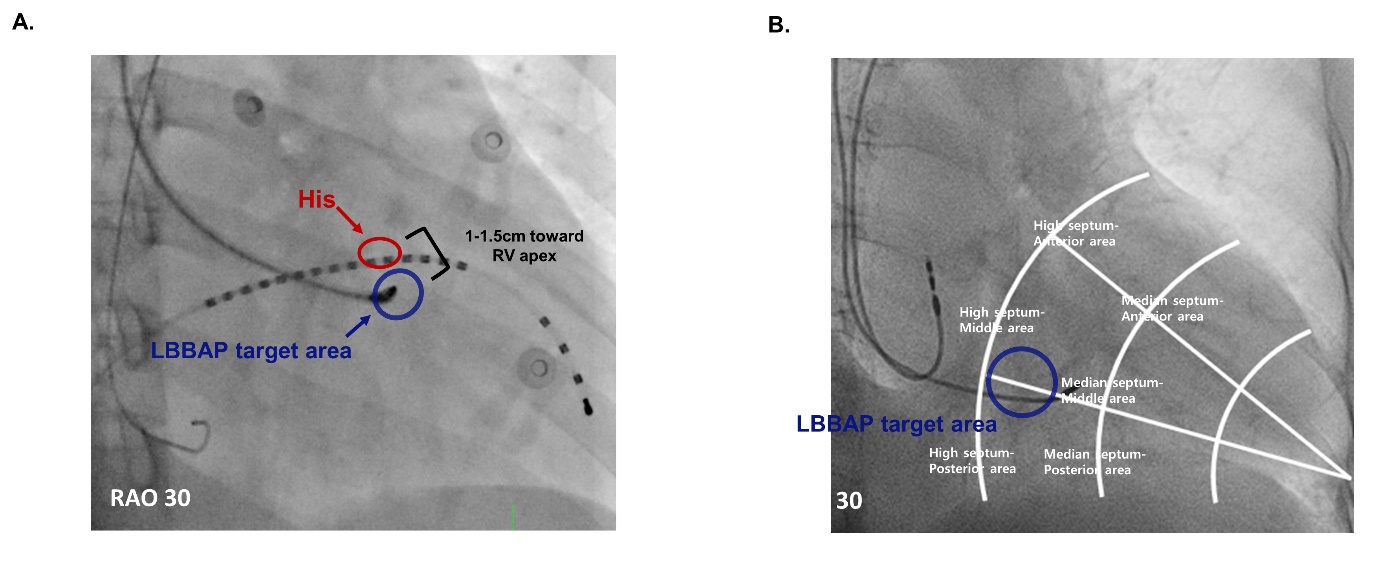
**

His area tagging method (A) and simplified nine-segment method (B) are presented.

LBBAP, left bundle branch area pacing; RAO, right anterior oblique; RV, right ventricle.

**Supplementary Table S1.** Trends in paced QRS duration according to baseline QRS morphology

|  | **Paced QRS duration (ms)** | | | | |
| --- | --- | --- | --- | --- | --- |
|  | 1 month | 3 months | 6 months | 12 months | P for trend |
| **All patients** | 127.1 ± 20.0 | 130.6 ± 27.4 | 126.8 ± 21.2 | 131.2 ± 23.8 | 0.322 |
| **Baseline QRS morphology** |  |  |  |  |  |
| Narrow QRS | 120.8 ± 21.9 | 126.8 ± 27.5 | 118.4 ± 22.0 | 122.3 ± 25.0 | 0.588 |
| RBBB | 137.6 ± 10.7 | 127.8 ± 37.4 | 130.9 ± 18.2 | 147.1 ± 17.9 | 0.028 |
| LBBB | 129.6 ± 20.1 | 137.4 ± 16.3 | 127.2 ± 19.6 | 130.0 ± 18.8 | 0.806 |
| Bifascicular block | 144.0 ± 11.3 | 139.3 ± 22.7 | 133.0 ± 1.4 | 135.5 ± 28.1 | 0.976 |
| Trifascicular block | NA* | NA* | NA* | NA* | NA* |
| IVCD | 115.0 ± 22.5 | 142.5 ± 18.9 | 143.7 ± 19.7 | 132.8 ± 20.5 | 0.437 |

Values are presented as mean ± standard deviation.

Serial changes in paced QRS duration (ms) are presented from 1 month to 12 months post-left bundle branch area pacing.

IVCD, intraventricular conduction delay; LBBB, left bundle branch block; ms, millisecond; RBBB, right bundle branch block.

*NA, not applicable as there were no patients with trifascicular block in the study population.
